# Activity of Superoxide Dismutase and Catalase in Fenugreek (*Trigonella foenum-graecum*) in Response to Carbendazim

**DOI:** 10.4103/0250-474X.62248

**Published:** 2010

**Authors:** R. Sangeetha

**Affiliations:** Department of Biochemistry, Vels University, Chennai-600 117, India

**Keywords:** Carbendazim, catalase, fenugreek, superoxide dismutase

## Abstract

Fenugreek (*Trigonella foenum-graecum*) is an annual herb, used as a spice and traditionally as medicine. Fenugreek finds its uses in treating hyperglycemia, hyperlipidemia and disorders of gastro-intestinal and cardiovascular systems. Fenugreek cultivation in India is affected by fungal diseases like root-rot and damping-off and fungicides like carbendazim are used to overcome these infections. Fungicides play both positive and negative role in plants; fungicides protect plants from diseases and also exert oxidative stress simultaneously. This report is on the response of antioxidants, superoxide dismutase and catalase in fenugreek seeds and plants treated to different concentrations of carbendazim.

Fenugreek (*Trigonella foenum-graecum*) is an annual herb and a medicinal plant. The uses of the seeds and leaves of fenugreek are diverse. They are used as spices in food preparations to enhance or impart flavour. Fenugreek seeds are good sources of protein, fat, minerals and dietary fibre[1]. The notable chemical constituents of fenugreek are proteins rich in lysine and tryptophan, flavonoids such as quercetin, trigonelline, saponins, phytic acid and polyphenols[1,2]. The well documented therapeutic uses of fenugreek are its hypoglycemic and hypolipidemic activity[3,4]; fenugreek seed extracts normalize the enhanced lipid peroxidation and relieve oxidative stress by providing antioxidants in diabetic rats[5]. Fenugreek also serves to protect the gastrointestinal[6] and cardiovascular systems[7].

Fenugreek requires a relatively cool climate for its propagation and is highly susceptible to fungal infections. The main diseases that affect fenugreek are foot-rot and damping-off disease in India; leaf spot and powdery mildew are diseases reported to affect fenugreek in other parts of the world[8]. Many different types of fungicides have been evaluated and used to control fungal infections and carbendazim was found to be very effective in controlling foot-rot and damping-off diseases[9]. Fungicides though serve to prevent the incidence of fungal infections and improve quantitative yield, deteriorate the quality of the seeds by altering the composition of seeds and cause undesired biochemical and metabolic changes[10]. This study was aimed at analyzing the effect of carbendazim at different concentrations on germinating fenugreek seeds and plantlets. The response of the antioxidant defenses namely superoxide dismutase (SOD), catalase (CAT) was evaluated and reported in this paper.

Dry fenugreek seeds were purchased from a local grocery. The fungicide used was carbendazim (Bavistin) and was bought from a local chemical shop. All the chemicals used for analyses were of analytical grade. Nitroblue tetrazolium and riboflavin was obtained from Himedia, India.

The seeds were washed to free from dust and rinsed with 1% mercuric chloride for sterilization. Ten grams of seeds were soaked in distilled water for the control and for treatments, the seeds were soaked in 0.05, 0.1 and 0.3% carbendazim overnight. The concentrations of carbendazim used were according to the suggestions made for fenugreek cultivation by Farrooq *et al*[11]. The control and treated seeds were then washed with distilled water and placed in a Petri dish layered with moistened paper towels. The plates were kept in dark and the paper towels were kept moist to aid seed germination. The assays were performed for the next five days as three independent experiments.

The sterilized seeds were raised in earthen pots and were allowed to grow. On the 20^th^ day, a foliar application of 0.05, 0.1 and 0.3% carbendazim was given to the different treatment groups. The next foliar application of carbendazim was after 30 days. The control plants grew only on distilled water. The assay was performed for 5 days after the second foliar application as three independent experiments.

The seed coats were removed and the intact cotyledons were homogenized using chilled 0.1M sodium phosphate buffer (pH 7.4) to prepare a 10% homogenate. The homogenate was centrifuged at 10 000 rpm at 4° for 10 min and the supernatant was used for assays. In case of foliar spray, leaves were excised fresh and homogenized as mentioned above. The activity of SOD was measured by the method of Kakkar *et al*[12]. Catalase activity was determined by the method of Aebi[13]. Total proteins present were assayed by the method of Bradford[14]. Zymogram analysis of SOD was performed by the method of Beauchamp and Fridovich[15].

The percentage of germination was the same with the control seeds and with the seeds treated with 0.05% carbendazim. The seeds with 0.1 and 0.3% carbendazim treatment exhibited low germination percentage. The root and shoot length of the control plants and the treated plants was similar. Thus treatment with carbendazim affected germination physiology only.

The level of the antioxidants, SOD and CAT decreased gradually in all the seeds during the first five days of germination. The activity of SOD and CAT was significantly higher in the seeds treated with 0.05% carbendazim than in the control seeds (p>0.01). The seeds with the other two higher concentrations of carbendazim had low SOD and CAT activities and the decrease was statistically significant (p>0.01). A similar response was observed when carbendazim was applied as a foliar spray. A decrease by 40% and more in the activities of SOD and CAT was observed in the plants which received 0.1% and 0.3% carbendazim (figs. 1 and 2). Fungicides at high concentrations protect plants but exert oxidative stress[10]. The decrease in the levels of SOD and CAT in the presence of 0.1% and 0.3% carbendazim could be attributed to the increased utilization of these antioxidants to combat the reactive oxygen species produced excessively during the oxidative stress.

**Fig. 1:**
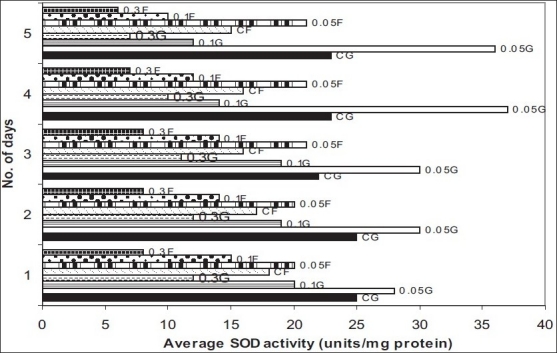
Effect of carbendazim on activity of SOD in germinating fenugreek seeds and plants The values represented are the average of three independent experiments. Seeds and plants treated with 0.05% carbendazim (0.0G and 0.05F, respectively) exhibit significantly (p>0.01) increased SOD activity when compared to control seeds and plants (CG and CF, respectively). Treatment of seeds and plants with 0.1 and 0.3% carbendazim (0.1G, 0.1F, 0.3G, 0.3F) decreased SOD activity (p>0.01).

**Fig. 2:**
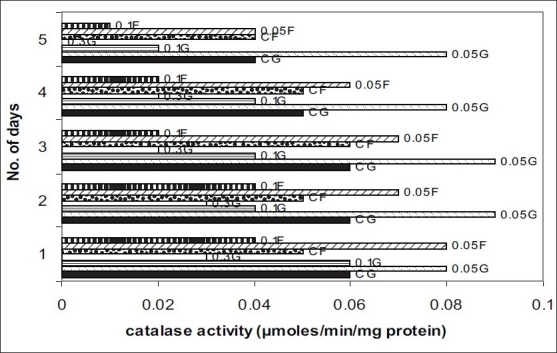
Effect of carbendazim on activity of catalase in germinating fenugreek seeds and plants Catalase activity is the average of three independent experiments. Seeds and plants treated with 0.05% carbendazim (0.0G and 0.05F, respectively) exhibit significantly (p>0.01) increased CAT activity when compared to control seeds and plants (CG and CF, respectively). Treatment of seeds and plants with 0.1 and 0.3% carbendazim (0.1G, 0.1F, 0.3G, 0.3F) decreased CAT activity (p>0.01).

The increase in both the antioxidants in seeds and plants exposed to 0.05% carbendazim was interesting as the response was reverse to that obtained with higher concentrations. Zymogram analysis of SOD was further performed to confirm the above stated results. The activity staining of SOD on polyacrylamide gels substantiates the results obtained by spectrophotometric analysis. The activity of SOD in germinated seeds treated with 0.05% carbendazim was higher than that in control seeds while the activity of SOD in seeds treated with 0.1% and 0.3% carbendazim was not detectable in the acrylamide gels (fig. 3). There are many reports on fungicides belonging to the triazole groups which can improve plant defense systems[16,17]. The findings on the efficiency of carbendazim to enhance plant antioxidants are very few. Smita and Nayyar[18] have proved that carbendazim can alleviate water stress. This study thus shows that carbendazim can improve plant defense mechanisms at low concentrations and thus improve the quality of the seeds and plants which are consumed to overcome oxidative stress associated with disorders like diabetes. Briefly, carbendazim which exerts oxidative stress at high concentrations can improve seed vigour when used at low concentrations. The actual mechanism behind this is considered for investigation in future.

**Fig. 3:**
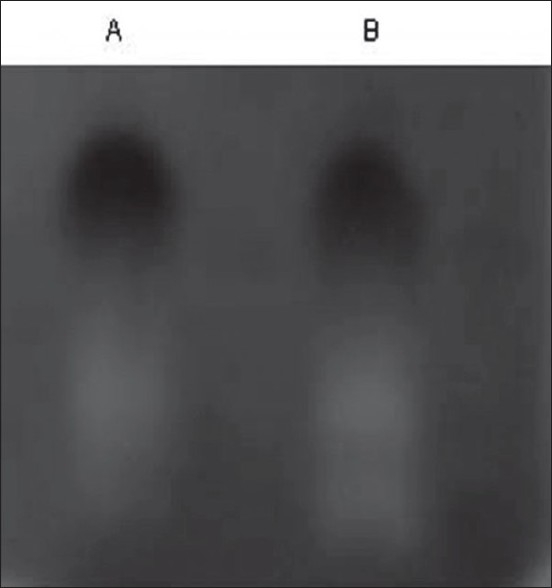
Zymogram analysis of SOD in germinating fenugreek Lane A- SOD activity in germinating control seeds; Lane B- SOD activity in geminating seeds treated with 0.05% carbendazim. Activity staining performed with extracts obtained on the 3^rd^ day of germination
